# Navigating lock-ins for adaptation: A case study of grid capacity planning in the Dutch energy transition

**DOI:** 10.1007/s11625-025-01793-6

**Published:** 2026-01-21

**Authors:** Hazal Deniz Kaya, Martijn Leijten, Daan Schraven, Paul W. Chan

**Affiliations:** 1 Faculty of Architecture and the Built Environment , Delft University of Technology , Julianalaan 134, 2628 BL Delft, The Netherlands; 2 Faculty of Technology, Policy and Management, Delft University of Technology, Jaffalaan 5, 2628 BX Delft, The Netherlands

**Keywords:** energy transition; electricity infrastructure; grid planning; sustainability transitions

## Abstract

Lock-ins are typically seen as barriers to sustainability transitions, particularly in the energy sector, where they can impede the radical changes needed for decarbonization. This study, however, argues that lock-ins can also act as catalysts for innovation within grid operators’ operational practices. Focusing on a Distribution System Operator (DSO) in Central-North Netherlands, the research explores how material, behavioural, and institutional lock-ins influence grid capacity planning for energy transition. Using a qualitative system dynamics methodology, the study reveals how these lock-ins contribute to grid congestion and delayed infrastructure development, but they also create pressure for adaptive change through three key mechanisms: (i) reframing questions, (ii) reorienting synergies between actors, and (iii) rediscovering solutions. These efforts have shifted the organization’s focus from reliability to flexibility, restructured internal operations to manage congestion, and enhanced collaboration with customers, regional authorities, and other energy system actors. However, challenges remain, including the need for a more innovation-driven organizational culture, stronger cooperation between regional and national grid operators, and greater public engagement in congestion management. By framing these findings within the tactical level of sustainability transition management—where strategy meets operations—this study demonstrates how electricity infrastructure can respond to lock-in conditions through adaptive strategies that turn systemic constraints into drivers for innovation, fostering more sustainable and resilient energy systems.

## 1 Introduction

The power grid, initially designed for centralized electricity distribution, faces growing strain from the rapid integration of distributed energy resources (DERs) and increasing demand from electrified heating and transport systems (Zarco-Soto et al. 2021). In many countries, the intermittent nature of renewables—combined with rising electricity demand—has created significant operational challenges, including voltage instabilities, reduced infrastructure lifespan, and risks of supply disruptions. Among these, grid congestion, when the required power exceeds the technical limits of cables and equipment, has become a particularly pressing issue. Traditionally, grid operators have responded to this capacity challenge by reinforcing their assets; however the magnitude and pace of capacity expansion required in many countries has become so substantial in many countries, including Netherlands (Netbeheer Nederland 2023), Germany (Bundesnetzagentur 2021), and United Kingdom (ESO 2024), combining with shortage of technicians, limited space, and financial constraints (Verhoeven et al. 2022). This situation calls for more resilient grid strategies that go beyond traditional reinforcement—such as energy storage, improved system integration, and demand-side management (Kabeyi and Olanrewaju 2023; Haley et al. 2020). However, current regulatory frameworks and planning processes often worsen congestion and impede broader energy transition efforts.

In this context, energy policies increasingly highlight the critical role of Distribution System Operators (DSOs)—entities responsible for managing the electricity distribution grid and ensuring reliable power delivery from transmission networks to end-users. As regulated infrastructure providers embedded in established routines and governance arrangements, DSOs can be understood as regime actors—traditionally passive, with a main focus on ensuring reliability through asset maintenance and expansion. However, DSOs are now expected to play an active role in managing grid stability, integrating renewables, and enabling decentralized energy systems (Pereira et al. 2020). While prior studies have examined legal (Edens and Lavrijssen 2019) and economic (Pena-Bello et al. 2023) barriers to this transition, the socio-technical dynamics influencing DSO’s ability to restructure the grid infrastructure remain underexplored. This research adopts a holistic approach, applying the theoretical lens of socio-technical lock-ins—framing the energy grid as a socio-technical system (Bolton and Foxon 2015)—to identify leverage points in how DSOs navigate grid capacity planning and sustainability strategies within existing governance frameworks.

Sociotechnical lock-ins, a widely discussed concept in energy infrastructure literature, refer to situations where technologies, systems, or policies become entrenched and resistant to change, even when better alternatives emerge. Unruh (2002) introduced this concept in the context of energy systems, showing how technological, institutional, and social dependencies reinforce fossil fuel reliance. Voß and Kemp (2006) further defined lock-ins as structural configurations that constrain future development pathways. In the context of sustainability transitions, it is critical to anticipate and assess the long-term systemic impacts of current actions to avoid lock-ins that hinder sustainable development.

Energy infrastructures, as large technical systems, often resist change due to their complexity and stakeholder interdependencies, creating self-reinforcing feedback loops that strengthen lock-ins (Goldstein et al. 2023). In energy transitions, lock-ins are frequently framed as carbon lock-ins, where infrastructural, institutional, and behavioural dependencies perpetuate fossil fuel systems, making them hard to displace (Seto et al. 2016). Discursive lock-ins also play a role, as political narratives and risk perceptions shape energy choices (Buschmann and Oels 2019). Even low-carbon policies, such as feed-in tariffs, may unintentionally reinforce existing regimes through mechanisms like rent-seeking (Nordensvärd and Urban 2015). Consequently, lock-ins are widely studied in energy transition literature as barriers to the radical changes needed for sustainability, shaping future transition pathways (Arapostathis and Pearson 2019). However, there is a notable gap in exploring how lock-ins might be adapted or leveraged to enable innovation, rather than solely focusing on overcoming them through radical transformation. Furthermore, much of the literature emphasizes historical decisions and future pathways, often neglecting the operational perspective of infrastructure planning.

Operating as regulated geographic monopolies, DSOs face inherent physical and operational constraints, leaving them vulnerable to fluctuations and uncertainties from the liberalized production and consumption sides of the market. This creates a ‘locked-in’ position of this regime actor that restricts their operational flexibility, making the theoretical lens of lock-ins particularly relevant for analysing distribution grid infrastructure. By applying this lens, we can better understand how DSOs navigate sustainability transition challenges while managing their internal sectoral entrenched constraints, especially as these increasingly clash with external pressures such as the promotion of renewables, electrification, and decentralization—each introducing new field logics that challenge existing routines.

This study addresses a critical gap in the literature by applying the concept of socio-technical lock-ins to the distribution grid—a previously underexplored area—using the Netherlands as a case study. It investigates how grid operators navigate lock-in challenges in grid capacity planning for decarbonization, shifting the focus from viewing lock-ins purely as barriers to understanding how DSOs adapt to and innovate within these constraints. The study will address the following research questions:

- **RQ1-** How do lock-ins influence distribution grid infrastructure capacity planning for decarbonization?
- **RQ2-** How are DSOs navigating lock-ins for innovative approaches, and what are the challenges and prospects associated with their strategies?

Using qualitative system dynamics modelling, the study identifies causal relationships and feedback loops that reveal lock-in mechanisms shaping grid operations, providing a holistic understanding of system complexity and socio-technical interdependencies critical for sustainability transitions (Gooyert et al. 2024). By mapping these structures and high “leverage points”, it pinpoints interventions to overcome policy resistance and accelerate the transition (Sterman 1994). In this paper, we use the term “leveraging lock-ins” to describe how actors engage with and respond to systemic constraints in ways that stimulate innovation, inter-organizational learning, and adaptive planning, even when structural barriers persist. Therefore, the research examines factors driving alignment or divergence in grid planning and how path dependencies influence attitudes toward sustainable innovation, challenging the perception of lock-ins as purely obstacles.

In Section 2 , the background will be presented on socio-technical lock-ins in organizational studies, and Section 3  will delve into the methodology of the research. Section 4.1   will first look into the different lock-ins that affect the grid operations towards energy carbonization goals, and Section 4.2  will concentrate on the responses/adaptation patterns of the DSO around the locked-in mechanisms. According to the challenges and prospects behind these patterns, Section 5  will present a discussion and policy implications, and Section 6will 4.1 Lock-ins of grid capacity planning for energy transitionoffer the conclusions and recommendations.

## 2 Theoretical background

### 2.1 Positioning the distribution grid operator in transitions

Socio-technical systems refer to physical technological artefacts that are both socially constructed and society-shaping (Hughes 1987). These systems include not only technical components but also the people, organizations, and institutional frameworks that shape their development and operation. Infrastructure systems, in particular, are socio-technical systems in which actor networks, institutional arrangements, and interdependencies are as integral as the physical assets (Kaijser 2017). Socio-technical transitions involve long-term changes in these systems—such as energy, water, or mobility (Hölscher et al. 2018). The MLP offers a way to understand long-term transition processes (Geels and Schot 2007) and is widely applied to analyse socio-technical system dynamics (Köhler et al. 2020; Lucas-Healey et al. 2022). It frames transitions as non-linear processes shaped by interactions across three levels: the landscape of exogenous pressures such as cultural norms, political, and economic shifts; the regime, where dominant actors, rules, and practices stabilize systems (Avelino and Wittmayer 2016); and niches, protected spaces where alternative technologies and radical innovations emerge and potentially challenge existing regimes. Transitions unfold through shifts in regime configurations and broader structural change across technologies, markets, and institutions. Yet, strong interdependencies across these levels often reinforce stability and create systemic lock-ins that hinder transformation (Grin et al. 2010) 

Building on this perspective, recent transition research calls for greater attention to how regime structures are shaped and transformed through the strategic interplay of actors, such as incumbent infrastructure providers, with a focus on who drives system innovation and how it emerges from within the regime (Fuenfschilling and Binz 2018; Grin 2020). Rather than treating regimes as static or focusing only on technological substitution, this line of inquiry emphasizes the role of actors embedded in regimes and their role on sectoral transition dynamics.

In this study, we adopt an actor-centred view by conceptualizing the DSO as a regime actor within the sociotechnical system of electricity distribution. As regulated monopolies, DSOs operate under formal regulations, institutional routines, and entrenched technical systems—features that exemplify regime characteristics such as path dependency and limited flexibility for experimentation (Kungl and Geels 2018). Although DSOs are critical for integrating decentralised renewables and rising electrified demand at the distribution grid, they have received relatively little attention in transition research, which has tended to focus on high-voltage transmission, production, or supply actors (Steenhuisen and de Bruijne 2015; Sonnsjö 2024). Rather than applying the MLP as an analytical framework, we use the regime-level positioning of the DSO to examine how its established roles, routines, and norms are challenged by landscape pressures and the shift toward a renewable-based regime—and how the organisation responds to these tensions. To understand how such tensions are manifested and navigated, the next section introduces socio-technical lock-ins as a lens to explore both regime constraints and the potential for adaptation in energy transitions.

### 2.2 Socio-technical lock-ins in energy transition

Lock-ins arise from a complex interplay of factors, including existing infrastructure (representing sunk costs), formal institutional processes, established markets, capital availability, power relations (elites maintaining the status quo), consumption patterns, values, preferences, and dominant discourses (Goldstein et al. 2023). In the context of energy transitions, these sources encompass the costs of uncertainty over incumbent technologies (Klitkou et al. 2015), incumbent know-how, expertise, existing state-industry relations, consumption patterns, or consumer environmental values (Trencher et al. 2020). These sources of lock-in are not static but rather dynamic and deeply intertwined (Simoens et al. 2022). For instance, the regulatory obligation to provide universal grid access (institutional) reinforces habitual expectations of unlimited electricity (behavioural), while simultaneously placing pressure on aging infrastructure (technological). Building on this literature, this study adopts the following categorization to analyse lock-in mechanisms:

**Table 1:** Overview of material, institutional, behavioural sources of lock-in mechanisms [adopted from Simoens et al. (2022); Seto et al. (2016), Klitkou et al. (2015), Arthur (1994)]

Category	Interlocking Mechanism	Description	Adopted from
Technological	Economies of Scale	Lower unit costs from increased production make established technologies more attractive for new investments.	Arthur (1994)
	Technological learning effect	Over time, products improve and costs decrease as production experience and knowledge grow, giving incumbents a significant advantage over new technologies.	Arthur (1994)
	Adaptive Expectations of Technology	Wider technology adoption reduces uncertainty about quality, performance, and durability, reinforcing further adoption.	Arthur (1994)
	Return on Investment (Sunk costs)	The long life of physical infrastructure makes it difficult and costly to change.	Seto et al. (2016)
Institutional	Collective action (legislative, regulatory, legal)	Institutional arrangements depend on coalitions and networks, becoming harder and costlier to change as they entrench.	Klitkou et al. (2015)
	Institutional learning effects	Institutional learning improves coordination and adaptive expectations, reinforcing adoption while embedding complexity that makes change difficult.	Klitkou et al. (2015)
	Power asymmetries	Those in institutional power can reshape rules to strengthen their position, further reinforcing the system that benefits them.	Klitkou et al. (2015); Seto et al. (2016)
Behavioural	Habituation	Habits and routines are automatic and unconscious, reinforcing established practices.	Seto et al. (2016)
	Risk avoidance	Behavioural changes involve risk and uncertainty, slowing new practices.	Seto et al. (2016)
	Social structure	Established practices shape and are shaped by societal norms, which, as they shift or new technologies emerge, further drive adoption.	Seto et al. (2016)

While traditionally viewed as barriers to change, recent research highlights how lock-ins can also enable transformation (Goldstein et al. 2023). Public infrastructure, for example, often faces long-term lock-ins due to physical and regulatory constraints, where adaptive rather than disruptive solutions are needed to mitigate environmental impacts. In energy transitions, lock-ins and path dependencies can function as interlocking mechanisms within existing systems. Yona et al. (2019) show how policies and long-term contracts can lock-in renewables, creating positive feedback loops through sunk costs, increasing returns, and political reinforcement. Understanding these socio-technical lock-ins is crucial for navigating the shift from fossil fuels to renewables across scales.

Transformation, often seen as the opposite of lock-ins, can also arise from shocks and stressors that incentivize change. Meadows (1999) notes that small shifts in system elements, such as organizational structures or collective cognition, can trigger broader transformations. Similarly, Abson et al. (2017) argue that recognizing adaptation failures can open pathways for systemic change. Recent work indicates that lock-ins themselves may be strategically used to stabilise renewable energy transitions through power dynamics and socio-environmental incentives (Eitan and Hekkert 2023). This aligns with the idea of “flexibility” in infrastructure systems, where institutional path dependencies can act as positive lock-ins that support long-term adaptability (Helmrich et al. 2023; Buzási and Csizovszky 2023).

This research builds on these perspectives, focusing not on eliminating lock-ins but on understanding how their feedback loops—both constraining and enabling—can serve as stressors that create leverage points for sustainability transitions. As discussed earlier, lock-ins affecting regime actors such as DSOs operate both system-wide and internally within the organisation. Building on Table 1, we conceptualize socio-technical lock-ins as self-reinforcing mechanisms arising from technological, institutional, and behavioural interdependencies, shaping both the DSO and its external environment. As these lock-ins encounter increasing pressures from the energy transition, a mismatch arises between entrenched routines and evolving system demands. This resonates with Hughes (1987) notion of reverse salients, which describes imbalances that arise from uneven growth between system components in large technical systems. In our case, however, the imbalance is less about misalignment of system components and more about the gap between the rapidly growing demand of energy transition and the distribution grid’s limited capacity to accommodate this growth. This tension between the supply and demand compels the regime actor to adapt its practices. Accordingly, this study examines how organisational change interacts with wider sociotechnical transitions by exploring how lock-ins—typically viewed as sources of inertia—can also generate strategic friction that enables adaptation and innovation from within. The following table summarises the theoretical foundations on leveraging lock-ins in the literature.

**Table 2:** References on the positive role of Lock-ins in sustainability transitions

Lock-in Type	Positive Mechanisms	Key References
Technological Lock-ins	**Economies of Scale & Scope** (e.g., existing renewable energy regime accelerates further growth)	Klitkou et al. (2015)
	**Path-Dependent Innovations** (e.g., leveraging existing technologies and frameworks)	Klitkou et al. (2015), Eitan and Hekkert (2023)
	**Infrastructure Interrelatedness** (e.g., repurposing existing infrastructure for innovation)	Klitkou et al. (2015), Seto et al. (2016)
Institutional Lock-ins	**Institutional Learning** (e.g., knowledge transfer from regulatory, market, and legal frameworks)	Klitkou et al. (2015), Seto et al. (2016)
	**Institutional Alignment** (e.g., gradual shifts toward decarbonization, actor realignment)	Klitkou et al. (2015), Seto et al. (2016)
	**Regulatory & Policy Durability** (e.g., balancing stability with adaptability)	Yona et al. (2019)
Behavioural Lock-ins	**Norm Entrenchment** (e.g., reinforcing familiarity with sustainable practices, social acceptance)	Klitkou et al. (2015), Seto et al. (2016)
	**Habituation** (e.g., utilizing embedded routines)	Maréchal (2009), Klitkou et al. (2015)

This research began with an explorative phase, informed by socio-technical lock-ins literature (Table 1), to identify the main technological, institutional, and behavioural interlocking sources shaping feedback loops in distribution grid capacity planning. Building on these revealed dynamics, we then applied an abductive lens to examine their potential as catalysts for change, developing theoretical themes through iterative engagement with empirical data and existing literature (Table 2). A case study of a Dutch DSO’s capacity management informs this analysis. The next section outlines the case, data collection, and analysis methods.

## 3 Methodology

### 3.1 Case description

**Fig. 1:**
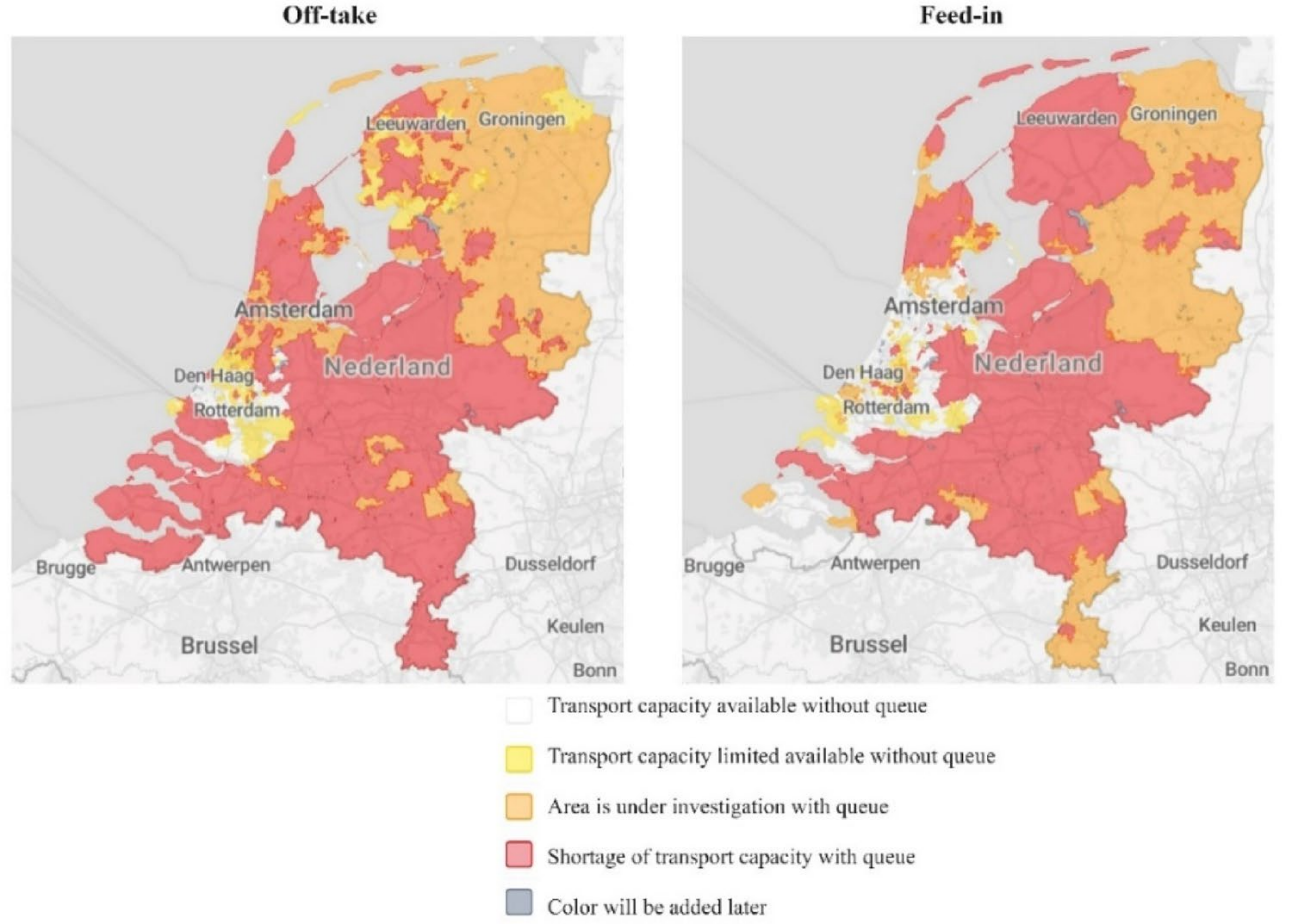
Congestion Map of the Netherlands (source: Netbeheer Nederland 2024)

The Netherlands aims to cut greenhouse gas emissions by 49% by 2030 and 95% by 2050, driving a rapid shift from fossil fuels to renewables (in Climate Agreement Dutch Parliament, 2019). Policies like the Coal Ban Act and investments in offshore wind and solar are accelerating this transition, with renewables projected to meet 53% of electricity demand by 2024 (Stat 2024). However, the electrification of transportation, heating, and industry has strained grid infrastructure, risking delays in the energy transition without significant upgrades (Planbureau voor de Leefomgeving 2023). Rapid urbanization and limited spatial capacity exacerbate these challenges, especially in densely populated regions like Utrecht, Gelderland, and Noord-Holland, where over 105 gigawatts of additional capacity have been requested (TenneT 2023). Despite substantial investments, grid congestion remains a critical barrier, as highlighted in the national congestion map, threatening the achievement of energy transition goals (Netbeheer Nederland 2024). (see Fig.1) 

In electricity systems, Transmission System Operators (TSOs) manage high-voltage grids and system stability, while DSOs oversee medium and low-voltage networks, delivering electricity to end-users and integrating distributed energy resources (DERs) (Uzum et al. 2024). Traditionally focused on subsurface infrastructure—laying cables, reinforcing grids, and building substations—DSOs are now transitioning to market facilitation, congestion management, and integrating decentralized generation into wholesale markets. Urban electrification and the rise of prosumers are amplifying distribution grid challenges, emphasizing DSOs’ critical role in managing congestion and DER integration (Verzijlbergh et al. 2017). However, DSOs operate under EU liberalization rules, which enforce ownership unbundling to prevent conflicts of interest (Electricity Directive 2009). The ‘copper plate approach,’ assuming unlimited grid capacity, promotes open access but overlooks physical constraints (Pfluger 2014), creating tensions between economic and environmental efficiency in a fragmented energy system (Kuiken and Más 2019). This misalignment between grid expansion and demand highlights how lock-ins hinder the energy transition, making Dutch DSOs a relevant case for studying the interplay between decarbonization goals and capacity challenges.

**Fig. 2:**
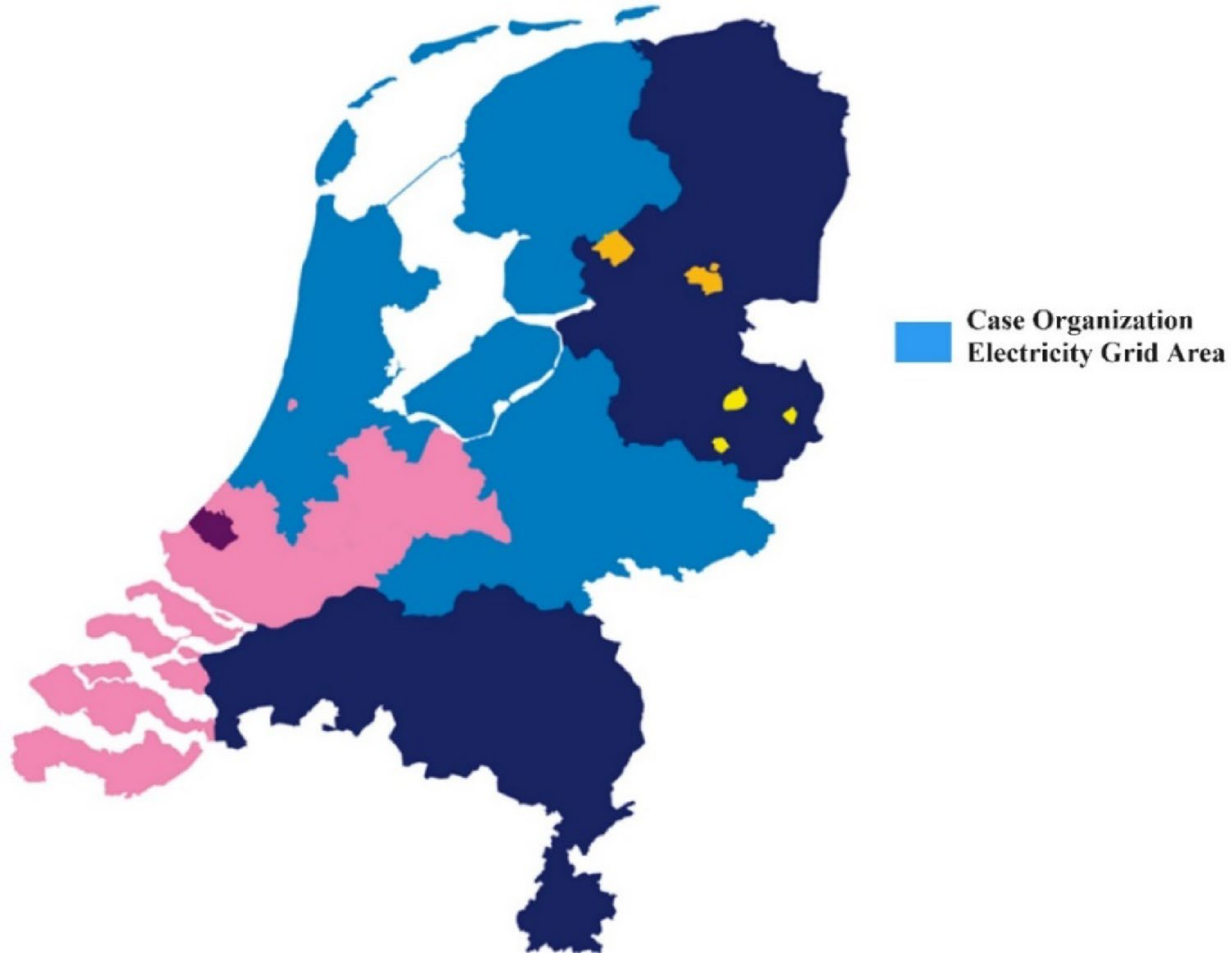
Case organization operation regions

This study adopts a single-case study to explore the underexamined phenomenon of how infrastructure providers adapt within lock-in conditions. The case organization is a leading Dutch DSO responsible for electricity and gas distribution across Gelderland, Noord-Holland, Amsterdam, Zuid-Holland, Friesland, and Flevoland (see Fig. 2). Its grids span 93,000 km (electricity), serving around 3.3 million consumers and businesses through over 5.7 million connection points. Operating in densely populated, industrialized regions with high energy demands, the DSO faces significant congestion challenges while navigating the energy transition, as evidenced by its operational area and congestion maps, making it a compelling case for examining the interplay between internal constraints and external transition pressures. Although the energy transition has been on the Dutch policy agenda for many years, recent national agreements, regulations, and subsidies have significantly accelerated the deployment of renewables and electrification. This rapid change is taking place in a context of limited spatial capacity and aging distribution infrastructure—conditions also found in many other European countries. These circumstances make the case a valuable example for examining how internal lock-ins, such as legacy assets or established routines, interact with growing external pressures, and how adaptive responses may emerge under urgent conditions.

Drawing on Nicholson et al. (2018), this paper aligns with what Corley and Gioia (2011) term a *revelatory* contribution—research that brings to light phenomena “we otherwise had not seen, known, or conceived.” Such work often engages in *problematization* (Grant and Pollock 2011), challenging prevailing assumptions in the literature. In the socio-technical lock-ins scholarship, these mechanisms are typically framed as barriers to sustainability transitions. However, our empirical findings reveal that the inability to match the pace of transition and its regulatory demands also prompted the case DSO to exercise agency and develop innovative responses under urgent conditions. The study thus reveals dynamics that, while context-bound, may resonate with other DSOs or infrastructure sectors facing similar capacity and transition challenges, particularly where rapid renewable integration clashes with the capacity of infrastructure systems.

The case study collects qualitative data through interviews and document analysis, focusing on the DSO’s grid capacity planning and management. This analysis identifies key lock-in mechanisms driving capacity challenges and the innovative practices they inspire, modelled using a qualitative system dynamics approach. The next sections detail the data collection and modelling methodology.

### 3.2 Qualitative data collection and analysis

**Fig. 3:**
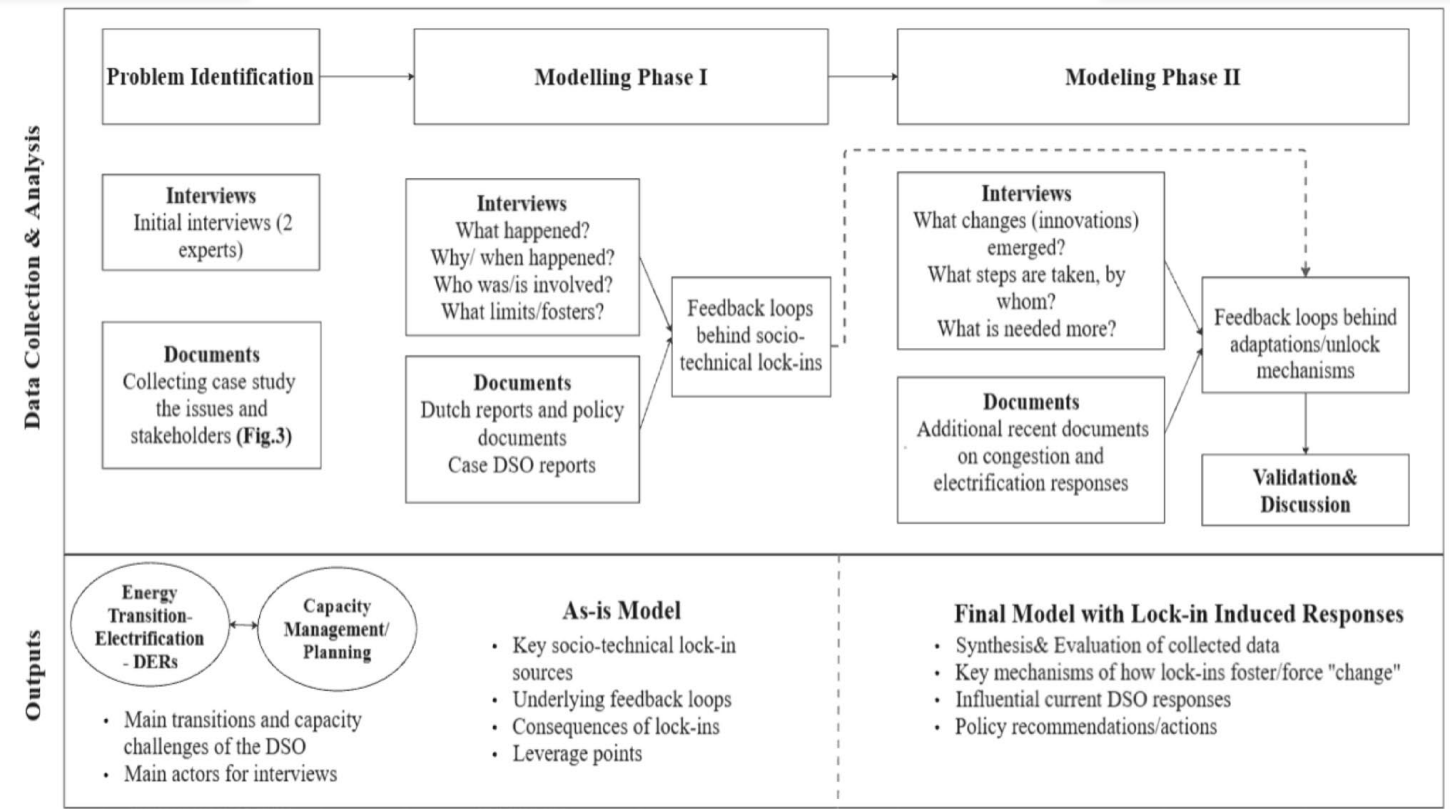
Research Methodology

This research primarily employs qualitative methods, including semi-structured interviews with the case DSO. The research methodology follows four steps: (1) problem identification, (2) qualitative data collection and analysis, (3) modelling phases, and (4) iterative validation and discussion, as can be seen in Fig. 3.

During the problem identification phase, initial interviews with two key experts—one overseeing corporate social responsibility and transition pathways, and the other managing regional operations—revealed the DSO’s main challenge: balancing growing electrification and distributed energy integration with grid capacity issues. Data was collected from documents and secondary sources, with initial experts referring the team to additional specialists in energy transition, capacity planning, congestion management, and grid operations at both strategic and operational levels (Table 3). Between December 2023 and March 2024, data were gathered through interviews and reviews of strategy papers, policy regulations, ACM reports, the national congestion program, and the climate agreement, offering insights into the DSO’s internal and external system lock-ins (see Table 4).

**Table 3:** List of interviewees and expertise

Int#	Responsibility	Years in Industry	Problem Identification	Semi-structured Interviews	Validation/Feedbacks
Int.1	Corporate Social Responsibility Director	10	X	X	X
Int.2	Regional Manager (North Holland)	5	X	X	X
Int.3	Lead Research Advisor- Digital Monitoring	15		X	
Int.4	Program Manager- Congestion Management and Market Innovation	17		X	X
Int.5	Senior Policy Advisor	15		X	X
Int.6	Consultant in Energy Transition	9		X	
Int.7	Innovation Development Lead	7		X	
Int.8	Team Leader in Grid Strategy	12		X	
Int.9	Technical Project Lead- Sustainable Grid Integration	10		X	X
Int.10	Innovation Consultant	10		X	
Int.11	Team Manager in Grid Operations	5		X	

**Table 4:** Reviewed documents for the case study

Name	Description	Organization	Pages	Date
Climate Agreement (Klimaatakkord)(Dutch Parliament, 2019)	National Climate Agreement of the Netherlands contains the main climate and energy policies.	Dutch Parliament	Related to Electricity (165–246)	2019
DSO Strategy Document	Key highlights, strategies, concerns, and steps of 2023	DSO	264	2023
DSO Annual Report (2023)	Current progress, issues, and steps in the face of energy transition	DSO	27	2023
E-Directive (2019) (contains E-Act 1998)	Provides the basis of the electricity market, law, and regulation	European Parliament and Dutch Government	75	Electricity and Act (1998), E-Directive (2019)
National Action Program- Grid Congestion (Government of the Netherlands, 2022)	Contains the strategies, measures, and steps for congestion action	Netbeheer Nederland, ACM, national and provincial governments, Energie Nederland	33	2022
The Netherlands Energy Policy Review (IEA, 2020)	Overview of the aims, goals, current energy issues, and policies	International Energy Agency (IEA)	Related to distribution grid activities	2020
Update of the National Energy and Climate Plan 2021–2030(Ministry of Economic Affairs and Climate Policy, 2024)	Climate and energy policy overview, recent targets	Ministry of Economic Affairs and Climate Policy	Related to distribution grid activities	2024

With consent, interviews (45–60 min) were recorded, transcribed, and analyzed alongside the documents as primary data. The analysis was structured to align with the study’s two research questions. To answer RQ1—how lock-ins influence distribution grid infrastructure capacity planning for decarbonization—we began with an explorative coding phase. This stage identified socio-technical lock-in mechanisms by tracing feedback loops and typologies informed by the literature (Table 1), highlighting how technological, institutional, and behavioural routines constrain current grid capacity planning. Interview questions were designed to uncover these constraints, revealing interlocking factors—mainly at the regime level, but also shaped by niche-to-regime interactions (e.g., renewable promotion) and landscape pressures (e.g., sociopolitical norms). The responses captured not only internal organizational constraints but also interdependencies with external rules, other regime actors (e.g., transmission grid operators, government), and wider societal dynamics. This approach allowed for a more comprehensive view of the lock-in mechanisms shaping the DSO’s activities.

To answer RQ2—how DSOs navigate these constraints in innovative ways—we adopted an abductive approach. This involved iterative engagement with both data and existing theory, particularly around the potential for lock-ins to induce adaptation and reconfiguration (Table 2). Rather than starting from a predefined framework, this phase allowed insights to emerge from the collected data and be refined in dialogue with relevant literature, as the merits of the abductive approach (Alvesson and Kärreman 2007). This process of uncovering theoretical themes related to how organizations respond to the consequences of lock-ins—particularly under urgent external pressures—was supported by ATLAS.ti and involved data reduction, display, conclusion drawing, and verification. Coding began with first-order codes representing adaptive responses to the constraints and mismatches created by interlocking lock-ins, such as strategic or institutional shifts. For example, an interviewee noted, *“We have refocused our strategy to expand the network*,* be flexible*,* and emphasize customer communication*,*”* with refocusing strategy coded as a first-order change. These were then grouped into second-order codes like changing KPI & strategy, which were further synthesized into three aggregate dimensions: (i) reframing questions, (ii) reorienting synergies, and (iii) rediscovering solutions, which will be explained in more detail in the Findings section.

The findings also informed the two-phase system dynamics modelling. The first phase identified key material, institutional, and behavioural sources of lock-ins and their underlying socio-technical dynamics, which defined the model parameters. The second phase explored how these constraints drive change by incorporating leverage points from interviews and documents. Together, the two phases produced the as-is and final qualitative models, in line with our two research questions. The next section details the system dynamics modelling process.

### 3.3 System dynamics (SD)

System Dynamics (SD) is a key method for analysing complex systems over time, particularly in uncovering feedback mechanisms that shape energy transitions and socio-technical interdependencies driving policy resistance (Stefes 2020; Gürsan et al. 2024). Originally introduced by Forrester (1987), SD maps causal relationships through feedback loops, helping to identify policy resistance (Ghaffarzadegan et al. 2011) and leverage points for intervention (Forrester 1987). It complements sustainability transition frameworks by providing a systems-thinking approach to understanding how feedback mechanisms influence transition pathways (Gooyert et al. 2016; Papachristos 2019). This aligns with the ‘problem structuring’ phase of transition management, where strategic, tactical, and operational actors collaboratively unravel system complexities (Loorbach 2010).

In this research, locked-in mechanisms of the DSO—shaped by historical events, technological choices, existing infrastructure, regulations, market rules, power relations, and organizational routines—are represented through causal loops, often reflecting feedback dynamics that influence capacity planning for the energy transition.

**Fig. 4:**
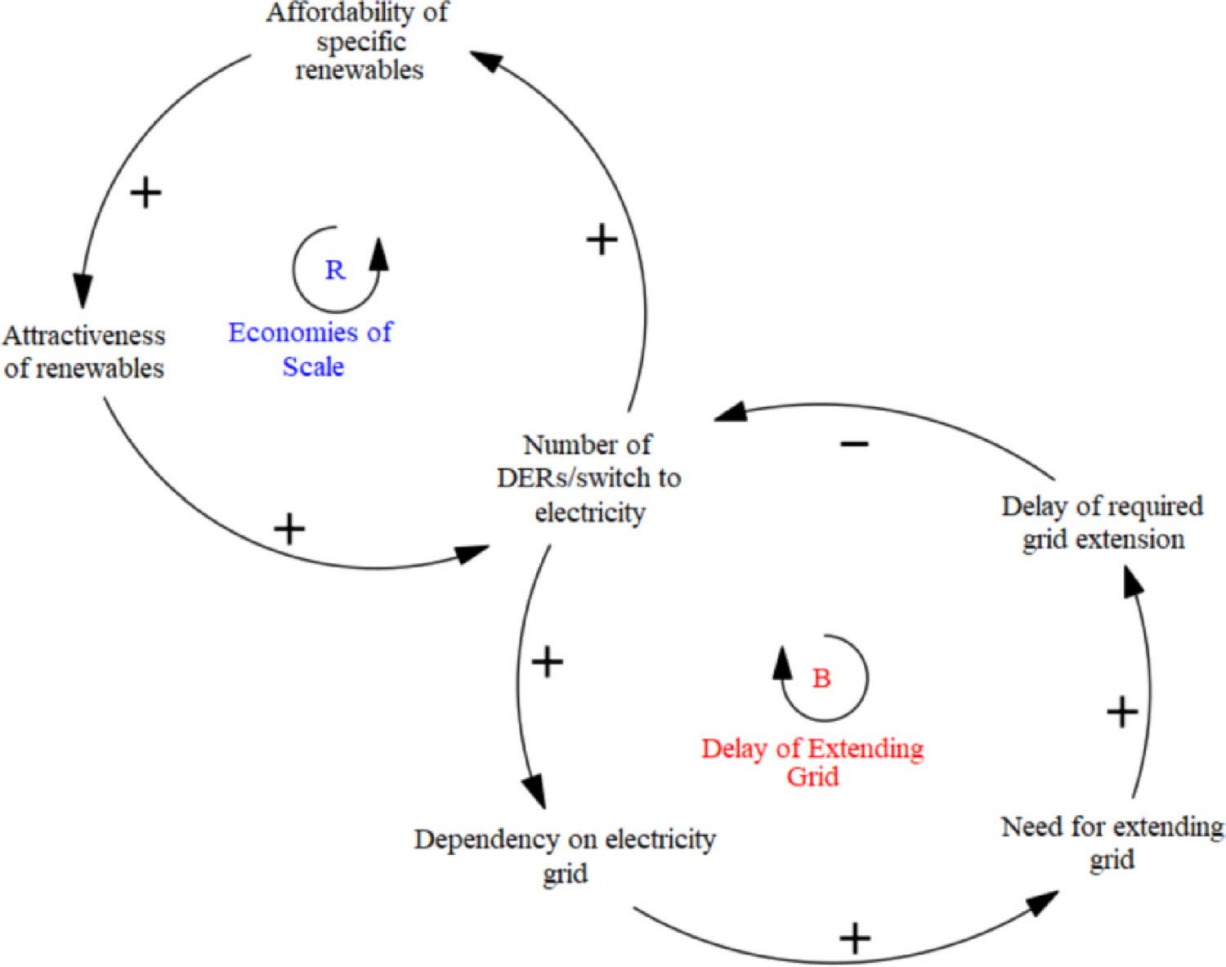
Example Causal Loop Diagrams

As shown in above Fig. 4, arrows represent causal links between variables, with ‘+’ indicating a positive relationship and ‘-’ a negative one. Feedback loops are labelled ‘R’ for reinforcing and ‘B’ for balancing. The model illustrates how the affordability of renewables drives lock-in through economies of scale, reinforcing adoption while increasing grid dependence. However, grid expansion delays create a balancing effect. Following Gooyert et al. (2024), qualitative analysis of interviews and secondary data informed the model by extracting key causal relationships, focusing on the DSO’s capacity challenges and innovation drivers. The first modelling phase visualizes lock-in complexities affecting the DSO, while the second phase integrates responses and ‘unlocking’ mechanisms (see 3.2), showing how lock-ins can drive change. Reinforcing loops around responses to grid scarcity highlight adaptation challenges and inform future policy recommendations.

## 4 Findings

In Section  4.1, through qualitative data analysis, we present the technological, institutional, and behavioural sources of lock-in in and around the energy planning system of the DSO, following the similar mechanisms stated in the transition literature (see Table 1), and examine how these mechanisms result in entrenched constraints and create a growing mismatch with the pace of the energy transition. In Section 4.2 , it will be explored how this very mismatch can generate adaptive responses—turning lock-ins into drivers of innovation, strategic realignment, and new forms of collaboration across the system to navigate sustainable energy transformations. All of the qualitative data from interviews and document analysis have also been indicated in the Supplementary Data.

### 4.1 Lock-ins of grid capacity planning for energy transition

Qualitative data analysis identified technological, institutional, and behavioural sources of lock-in that affect sustainable energy transition strategies. These were categorized and analysed with attention to their interlocking nature and the main consequences for energy transition goals The identified feedback loops, classified as reinforcing (R) or balancing (B), are further detailed in the text. These interlocking sources of lock-ins largely converged around the case organization’s central challenge, ‘grid scarcity’—which will guide the strategic leverage in the next section. While other historical and contextual factors also shape the grid operator’s transition efforts, this analysis focuses on interdependent constraints specifically in capacity planning to explore how they may be strategically navigated in the next modelling step.

#### Technological sources of Lock-in

Experts consistently emphasized that one of the key technological pressures on the grid stems from accelerating electrification—driven by the rise of decentralized renewable energy, the phase-out of natural gas, and growing electricity demand from sectors like heating, mobility, and industry. This rapid demand growth is reinforced by high energy prices and the falling costs of renewables, which together generate a reinforcing feedback loop **(R1)** between affordability and adoption (in Climate Agreement Dutch Parliament, 2019). However, this fast-paced transformation has created a severe imbalance between grid demand and system capacity. DSOs are legally obligated to accommodate both new production (e.g., solar and wind) and new loads (e.g., EVs and heat pumps) in an effective and cost-efficient way (E-Directive 2019), but infrastructure expansion cannot match the pace, causing persistent delays (**B1)** and congestion issues like overloading and voltage problems. As one interviewee put it, “The client is always faster than us, especially when subsidies support solar, wind, or EVs. The market grows enormously fast… the grid can’t grow that fast” (Int#4). What was once a relatively stable and predictable system has become a moving target: “Electrification is everywhere now, and the scale is massive. For medium-voltage it’s 100,000 km and for low-voltage, 145,000. So imagine, we need to double everywhere” (Int#8). This results in long waiting times for connections, balancing the pace of the energy transition **(B2).**

Grid infrastructure, originally designed for centralized generation, struggles to accommodate the rapid growth of distributed energy sources (EU Risk Preparedness in the Electricity Sector, 2019). “Low-voltage grids were never designed for households charging cars, heating homes electrically, and having solar panels at the same time” (Int#5). Another expert added, “We built this grid over 100 years, but now we need to double or triple its capacity in a decade—ideally in just two or three years” (Int#9). These trends extend beyond isolated bottlenecks, highlighting broader system expansion and integration challenges. The reliance on variable renewables adds volatility and peak loads: “More electricity use and more peaks on the grid, because renewable production is fluctuating” (Int#10), pointing to the challenge of intermittency.

Strengthening and expanding the grid is the most obvious solution, but space constraints slow down development, reinforcing technological lock-ins that limit renewable integration (**B4**). “Expanding the grid fast enough is difficult because space is scarce, and getting permits takes time” (Int#10). This challenge is particularly acute in urban and rural areas, where infrastructure must fit into already limited public space. Moreover, expanding the overburdened grid requires significant labour capacity: “now there’s overburdened grid capacity, and limited technical capacity among engineers and teams to expand it” (Int#6).

Another issue is the concentration of renewables in high- and medium-voltage grids (e.g., wind parks), leading to congestion and insufficient capacity for peak demand. “We initially assumed most solar would be installed on rooftops, but large-scale installations in rural areas are creating grid issues because production sites and demand centres don’t align” (Int#7). This also interlinks with the consolidation of specific technologies and creating inequalities between high- and low-voltage grids (e.g., households), potentially leading to low-voltage congestion in time (marked by delay in the SD model) and hindering a just energy transition **(B5).** Concerns were also raised about equity: “Some people will have solar panels and storage, reducing their reliance on the grid, while others, often lower-income households, will still face high electricity costs. How do we ensure they aren’t left behind in the energy transition?” (Int#3).

Economies of scale and learning effects in renewables technologies have accelerated diffusion but surpassed the grid’s physical capacity, leading to delays and congestion. All these technological sources are locking the system into a congested state, creating a capacity problem expected to last for years. Nearly 10,000 customers are already on waiting lists, and as one respondent put it: “I don’t think congestion will end anytime soon. For the next 10 to 15 years, it’ll likely persist.” (Int #9). Grid scarcity is widely recognized in national and internal documents, reinforcing the urgency for ‘unlocking’ innovations, which will be explored in Section 4.2  (see Table 5).

**Table 5:** Technological configurations challenging grid planning for energy transition

Technological Lock-in Mechanism		Explanation	Consequence/s
Economies of Scale		Rising energy prices increased electrification and DERs Unit cost of specific renewables decreased when output increased	Exponential growth of the distributed (decentralized) energy demand
Technological Learning Effects		The uncertainty of new renewable technologies decreased over time with accumulated knowledge over time	Locked-in technologies (Consolidation on specific sustainable technologies, especially in high-medium voltage grid)
Long-life Physical Infrastructure (Sunk-Costs)		Existing (available) energy grid capacity Existing dense spatial space	Delays of grid development Grid congestion- Waiting lists

#### Institutional sources of lock-in

Both international and national energy ambitions significantly shape the institutional configuration of Dutch energy grid planning. Electrification, driven by decentralized renewable energy sources, has become technologically locked in, largely as a result of national climate agreements and subsidy schemes. DSOs are legally mandated “to construct, repair, renew, or expand the networks, taking into account measures in the field of sustainable electricity or decentralized electricity production that can obviate the need to replace or increase production capacity” (E-Directive, 2019). However, the case study shows that the unbundled structure of European electricity markets, introduced in the late 1990 s, has made proactive planning increasingly difficult. Although transmission and distribution remain regulated, the liberalization of generation and supply (Electricity Act 1998) fragmented the system and limited coordinated innovation. As one interviewee noted: “As a liberal country, I understand the reasoning behind unbundling… it fostered competition and benefited customers. But many of today’s congestion issues might not exist if the sectors hadn’t been split. When we were one company, production planning could directly ask distribution or transmission, ‘Is there enough grid capacity for a new power plant or solar installation here?’” (Int#9).

Over time, unbundling formalized separation of roles and reinforced fragmented responsibilities, shaping institutional learning processes where actors protected their own interests under stable rules (Klitkou et al. 2015) rather than adapting collectively. For the DSO, this means carrying the burden of congestion issues rooted in production and supply dynamics. As one respondent put it: “As grid operators, we’re strictly regulated and required to solve these issues… but the same regulations don’t apply to the customers and producers using the grid” (Int#7).

Grid operators must make timely investments to accommodate growing energy demand, but Dutch DSOs are constrained by the requirement to manage networks in the “most efficient” way, often interpreted as economic efficiency (E-Directive 2019; Mulder 2016). This discourages proactive investments. “Regulation says we need to be as cost-effective as possible…with other DSOs, we are benchmarked against each other, whether we have unused grids or not” (Int#6). Another interviewee elaborated, “Even during 1997 Kyoto…we could have started laying thicker cables and building more substations, but the incentives were against it. The most cost-efficient operator would receive financial rewards…it didn’t make sense to pre-invest” (Int#8). Another added, “The regulative framework makes us ‘regulative captives,’ discouraging upfront investments and forcing us to delay enhancements” (Int#5), leading to investment lags that struggle to keep up with renewable energy growth, creating a balancing loop **(B3).**

The “copper plate” principle, which grants customers unrestricted grid connection rights, exacerbates congestion **(B6)** and shifts costs to grid operators. “A windmill can freely demand a connection, and we must comply. The copper plate principle gives everyone full freedom, but when congestion occurs, it’s always the grid operator’s responsibility” (Int#8). Another interviewee added, “Everyone connected to the grid can use their contracted capacity whenever they want… we must ensure we can accommodate everything” (Int#8). The “free-demand” model reinforces the illusion of unlimited grid access and the perceived need to reserve capacity, but as constraints grow, collaboration between grid operators and energy users must evolve—potentially breaking lock-ins. This model has also shaped customer expectations through institutional learning: a commonly held assumption that full capacity must always be guaranteed, regardless of actual use. As one interviewee explained, “We are bound by the regulations… and it created the common expectation—we have to provide any client with any capacity they ask. We have to provide it 24/7. For instance, they ask for 2 megawatts but at the end use only 1—we still need to ensure that they can use 2 when they need it” (Int#6).

Power asymmetries between government bodies, energy companies, and DSOs have intensified with the energy transition. DSOs’ demand-side measures quickly became political since “consumers are legally protected and don’t want DSOs restricting electricity use… the grid would melt if that were enforced” (Int#6). This complicates grid management, particularly at low-voltage levels where congestion is expected to rise. While DSOs manage medium-voltage congestion, stricter measures will be necessary at lower levels. However, such interventions remain challenging, as consumer freedoms are legally protected, reinforced by role separation and the “copper plate” principle—sustaining consumerism and behavioural lock-ins (see Table 6).

**Table 6:** Institutional configurations challenging grid planning for energy transition

Institutional Lock-in Mechanism	Explanation	Consequence/s
Regulative Arrangements	Regulatory frameworks prioritize cost-efficiency, discouraging pre-investment in grid expansion.	Delay of extending grid
Unbundling and Separation of Roles	Unbundled structure of grid operations, institutionalized separate roles, and reduced the uncertainty around the liberalized structure.	Lack of timely/informed investments
Copper Plate and Unlimited Demand	The “Copper plate” principle reinforces an institutional learning effect: the rule of unconstrained connection to the grid	Congestion
Power Asymmetries	Political actors use their authority to reinforce rules that limit restrictions on energy use, often framed as protecting individual freedom.	Congestion

#### Behavioural sources of lock-in

The technological and institutional configurations shape the attitudes of individuals and organizations toward their activities, reinforcing behavioural lock-ins. As interviews highlighted, subsidies have played a key role in promoting distributed renewables and increasing electrification demand. However, experts also raised concerns about rising consumerism and lifestyle changes driving the use of more electrical appliances. Consumption patterns are often automatic habits, reinforcing peak demand. “It’s like traffic—everyone wants to go home at the same time, just as everyone wants electricity at once, causing congestion” (Int#2). The growing electrification of housing, mobility, and industry is further intensifying demand. “Across all investment scenarios, consumption keeps rising. As more electric cars emerge and gas is phased out, electricity demand will continue to grow, putting increasing pressure on the grid” (Int#2).

The expansion of factories and large-scale businesses, especially in less-developed regions, puts significant pressure on the distribution grid. This reflects how business growth habits both shape and are shaped by technological and institutional lock-ins, such as existing infrastructure and the slow, costly grid expansion process. The ‘copper plate’ principle, which prevents DSOs from refusing service, further intensifies these pressures. Additionally, regulatory frameworks prioritizing cost efficiency create a risk-averse culture within DSOs, discouraging proactive grid development amid uncertain demand **(B3).** As summarized (see Table 7):

**Table 7:** Behavioural configurations challenging grid planning for energy transition

Behavioural Lock-in	Explanation	Consequence/s
Habituation- Attractiveness of renewables	Increasing consumption/integration of renewables, and associated behavioural demand patterns	Exponential growth of the electricity demand
Social structure- Consumerism norm	The growing energy use from new developments and consumption continuously reinforces itself.	Exponential growth of the electricity demand
Risk avoidance	Attitude of the DSO to upfront investments.	Delay of extending the grid/timely investments

**Fig. 5:**
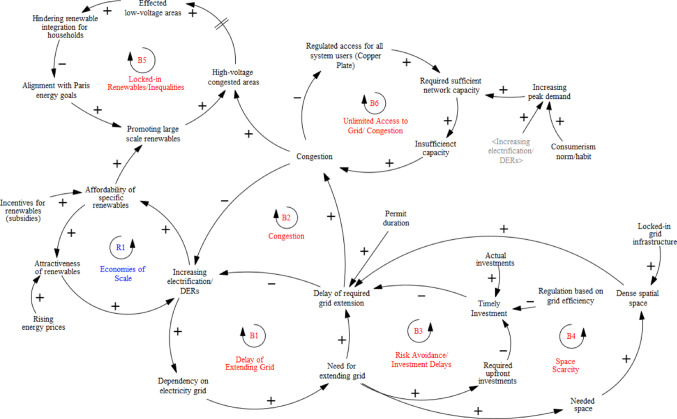
As-is Model representing the Lock-ins

The lock-ins influencing the case DSO’s operations were represented as a causal loop diagram (CLD) see Fig.5 , with iterative expert feedback. This system dynamics model illustrates the interdependencies between renewable energy adoption, grid congestion, and investment delays. Key reinforcing loops, like the affordability of renewables through economies of scale **(R1)**, drive further integration, while balancing loops such as congestion **(B2)** and grid extension delays **(B1)** highlight challenges in accommodating growing demand. Regulation based on grid efficiency makes planners ‘regulative hostages’, hindering the timely expansion of the grid **(B3).** The dense space and locked-in physical grid infrastructure further challenge the congestion **(B4)**, together with the timely permit process. Moreover, promoting large-scale renewables may create inequalities, limiting household integration as congestion spreads across voltage levels in time **(B5).** The “first-come, first-served” principle, combined with delayed grid development, causes congestion and limits “access for all” **(B6).** These loops reveal the complexities and trade-offs in scaling renewables while addressing infrastructure bottlenecks, showing how institutional and organizational factors shape grid planning, as validated by expert feedback on the SD model.

The research has identified key challenges and lock-in resistances hindering the DSO’s energy transition alignment. However, the case study reveals how the organisation is leveraging modelled lock-ins to adapt grid planning, with capacity constraints driving innovation. These adaptations and innovations are detailed in the following section.

### 4.2 Navigating lock-ins: how DSOs adapt and leverage constraints?

Interviews with practitioners from the case organization revealed that the locked-in situations analysed in the previous section have generated pressures for change in multiple ways. These adaptations show how the DSO responds to and works within entrenched constraints—what we frame as ‘leveraging lock-ins’—to develop new organizational, relational, and technological practices. These practices reflect how the DSO adapts to tensions between internal lock-in conditions— mentioned in the previous section, such as regulatory restrictions or infrastructural limitations—and external system changes, including landscape-level pressures (e.g., climate targets, electrification). These responses emerged through an abductive analysis—grounded in empirical data and informed by the literature discussed in Section 2.2 of how the identified lock-ins and their consequences shaped adaptive strategies to sustain the transition. We have categorized these effects into three key areas: (i) reframing the questions, (ii) reorienting the synergies between actors, and (iii) rediscovering solutions, as shown in Table 8.

While the mechanisms are presented as distinct, they overlap in practice and reflect different modes of responding to interlocking constraints. We interpret these patterns as forms of organizational adaptation within lock-in conditions, rather than isolated strategies. The following subsections will explore these aspects in greater depth through qualitative data analysis (coded through the interviews and documents Supplementary Data) and dynamic modelling of these responses, as represented in the system dynamics (SD) model.

**Table 8:** Example coding analysis of the adaptive responses (extended version in supplementary Data)

Raw data	Second-order codes: First-order codes	Aggregate code	MLP level	Related Theory
*“Ten to twenty years ago*,* we just installed cables and that was it. Now*,* as a distributing service operator*,* we’re shifting toward actively managing and controlling the grid.”* ***(Int#4)***	***Changing KPIs & Strategy***: Shifting priorities, updating KPIs, moving from expansion to control.	**Reframing questions**	Regime	N/A
*“A couple of years ago*,* we reorganized and created the System Operations department to focus on future grid and capacity management. … which aims to develop capabilities and products to support the grid and integrate customers on waiting lists.”* ***(Int#11)***	***Reorganizing Internally***: Creating new teams, roles, and capabilities internally		Regime-Niche Interface	N/A
*“I feel that the congestion problems we face today will actually help us reach this goal because everyone understands the problem and realizes we need to act.”* ***(Int #3)***	***Common Awareness***: Recognizing congestion as normal, shifting mindsets, and accepting limits		Regime	*Norm entrenchment * (Klitkou et al. 2015; Seto et al. 2016)
*“For a long time*,* people didn’t believe things could grow this fast—renewables stayed flat …with urgency once things really started moving.”* **(Int#8)**	***Organizational Urgency***: Growing urgency, faster decisions, rising risk awareness		Landscape → Regime	Abson et al. (2017)
*“Companies reserve capacity for future growth*,* but with growing shortages*,* this model is problematic—we can’t reassign unused capacity without disrupting their plans.”* ***(Int#6)***	***Raising New Questions on Capacity***: Reconsidering access rules, changing capacity allocation norms		Regime-Niche Interface (Reconfiguration)	*Norm entrenchment * (Klitkou et al. 2015; Seto et al. 2016)
*“It’s a complete shift from the internal focus to an external*,* customer-centric view. building connections with them” (****Int#3)*** *“We also need to educate customers—if they’re more informed*,* they may not request extra electricity…”****(Int#4)***	***Reconnecting Customer-DSO Governance***: Increasing regulatory flexibility for congestion management with the customer	**Reorienting** **Synergies**	Regime	*Institutional alignment * (Klitkou et al. 2015; Seto et al. 2016)
*“Historically*,* the TSO and DSO haven’t worked together on this. They’ve operated as separate entities*,* but now collaboration is beginning….”* ***(Int#10****)* *“we are more and more involved in active discussions in risk propositions of each other”* ***(Int#5)***	***Redefining TSO-DSO Coordination*** : Increasing alignment and real-time coordination across grid levels		Regime	*Institutional learning*,* actor realignment * (Klitkou et al. 2015; Seto et al. 2016)
*“Why can’t we expand the grid as fast as demand grows? Legal procedures*,* spatial issues*,* and complex coordination slow us down. Permits*,* planning rules*,* and every square meter is taken… make expansion difficult. We need better integration between development and the grid.” (****Int#6)***	***Reintegrating Spatial& Energy Planning***: land scarcity forcing integration; institutional spaces getting closer to the legal proceedings		Landscape → Regime	*Institutional learning*,* actor realignment * (Klitkou et al. 2015; Seto et al. (2016)
*On high-voltage grids*,* congestion impacts many customers*,* but that also means there are more opportunities to contract flexibility—often from larger consumers.”* ***(Int#5)*** *“…as businesses facing long wait times often find creative solutions.”* ***(Int#4)***	***Using the Existing Grid More Effectively***: Shifting demand, improving control, and using congestion to innovate	**Rediscovering Solutions**	Niche-Regime interface	*Infrastructure interrelatedness * (Klitkou et al. 2015; Seto et al. 2016)
*“We see positive trends in flexibility*,* like optimized EV charging at warehouses and peak reduction in PV installations—small adjustments can ease grid load with minimal financial impact.”* ***(Int#3)***	***Smart Charging &Smart Demand Control*** ***Solutions***: Flexible EV charging and time-based grid use		Niche-Regime interface	*Path dependent innovations; Economies of scale * (Klitkou et al. 2015; Eitan and Hekkert 2023)
*“The key question is where to locate storage to maximize available capacity for the market within grid constraints.”* ***(Int#7)***	***Storage Solutions***: Strategic and decentralized storage to ease grid pressure		Niche (emerging)	*Path dependent innovations; Economies of scale * (Klitkou et al. 2015; Eitan and Hekkert 2023)
*“Customers*,* especially generators*,* are already familiar with changing their operations based on electricity market prices.”* ***(Int#5)***	***Regulatory& Market-driven Solutions***: Leveraging market familiarity, price signals, and flexibility tools		Regime	*Market Habituation*,* Regulatory durability * (Maréchal 2009; Yona et al. 2019)

#### **Reframing questions: capacity management as an organizational challenge**

The qualitative data analysis revealed that the DSO’s locked-in challenges have reached a point of shared awareness, prompting a shift in the organization’s attitude. During the initial modelling phase, all participants identified delays in grid development **(B1)** and congestion issues **(B2)** as the primary sources of the current challenges, with congestion expected to persist for over a decade. This has led the DSO to refocus its strategy, establishing new departments for congestion management, flexibility market products, and customer relations. As Int#1 noted, “Since 2021, reliability is no longer the top KPI…we now have a complete flexibility department, exploring new market rules and incentives for efficient energy use.” Int#2 added, “The last 150 years were busy underground, but now building above-ground correlations is key…customers used to ask for cables; now we ask them to reduce usage—a different communication.” Int#11 explained, “We reorganized, creating a ‘system operations’ department to manage future grid capacity, but currently we lack the capacity to absorb all operational process changes in our department.” This reflects the DSO’s adaptive response to prolonged capacity constraints, reframing organisation strategies and structures to address challenges from energy transitions and grid scarcity. In doing so, it entrenches new norms of organizational responsibility while maintaining its historical role as a public entity that prioritizes societal value over economic considerations.

Congestion is now recognized as “the new normal” for the grid operator, as past locked-in decisions have led to a saturated and highly strained grid. This shared awareness reflects a behavioural lock-in, where established assumptions are now shifting, creating a sense of urgency that drives organizational adaptation. As Int#9 noted, “The congestion problems we face today will actually help us reach our goal because everyone understands the problem and realizes we need to act.” Grid scarcity, once seen as a constraint, is reframed as an economic certainty, ensuring full asset utilization and justifying future investments. Int#6 added, “In a way, grid congestion is economically beneficial…we know the grid will be used at 100% capacity. Having clients on a waiting list ensures our assets are fully utilized”.

Lock-ins have raised new questions about capacity, grid access, and allocation norms, as reflected in *The National Congestion Action Program*, developed in response to grid overburden in Limburg and North Brabant. The program highlights a shift from demand-driven production to supply-driven capacity management: “whereas previously demand determined production…now supply and grid capacity largely determine what demand the system can meet…the properties of the entire system are turning around—supply governs much more strongly now” *(*National Congestion Action Program, 2022).

Congestion is now hindering company growth and delaying consumer connection, by waiting lists which are incorporated into the SD model as a parameter negatively impacting the energy transition. As a result, many employees now focus on services improving efficient grid use, and question how to operate under the tensions of future planning and current grid scarcity. Additionally, in response to risk-averse behavioural lock-ins, the attitude is shifting toward upfront investments, recognizing that capacity will be utilized regardless, where the efficiency lock-in paradoxically aids under scarce capacity conditions.

#### **Reorienting synergies: building relational capacity with system actors**

Lock-ins, particularly grid congestion and reliance on grid development, have created new intersections between previously separate problem spaces, *reorienting synergies* among actors. Technological lock-ins, such as limited grid capacity, have increasingly aligned distribution grid planning with provincial, municipal, and infrastructure strategies. This convergence has strengthened collaboration, boosting confidence in grid investments and integrating energy planning as a crucial factor in urban and spatial development. As **Int#1** noted, “We have to think about where to increase investments…how we interact with companies, housing associations, municipalities, and provinces—a completely new way of working, with new challenges.” Int#10 added, “In the Netherlands, as in other countries, wind and solar farms are placed in rural areas with little grid capacity, requiring energy to be transported across the country.” For the same challenge, existing institutional knowledge across agencies has begun to align, driven by shared uncertainties and mutual learning.

**Fig. 6:**
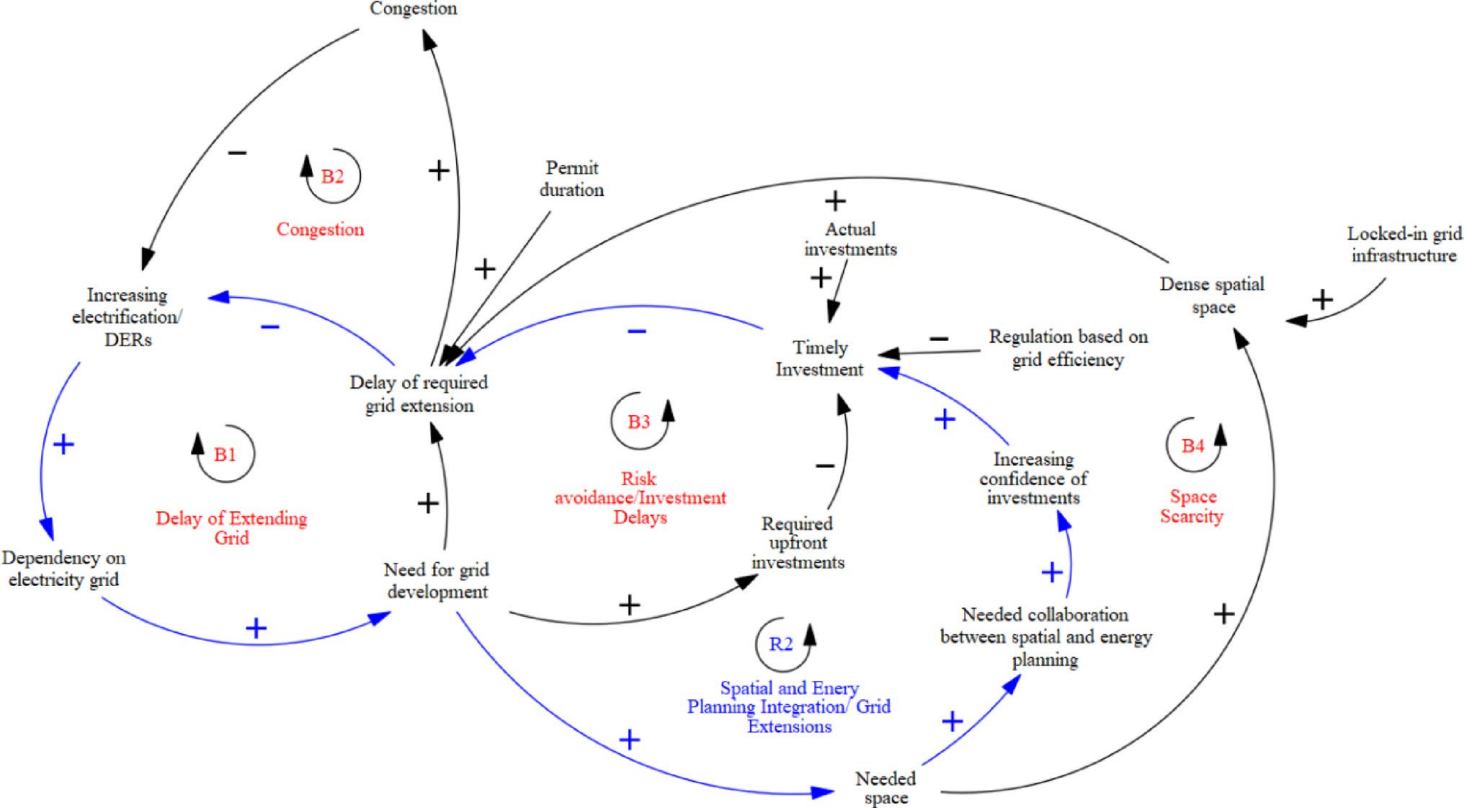
Reorienting Institutional Synergies Between Spatial and Energy Planning

The link between energy and urban planning, shaped by lock-ins, centres on network expansion and connection upgrades, driven by space scarcity. ‘Needed space’ was identified as a key parameter in the CLD, highlighting collaboration to boost investment confidence and reduce grid extension delays. With space for energy infrastructure increasingly limited, maintaining the energy transition’s pace requires faster, more certain space allocation—a challenge for municipalities that grid operators aim to support. This connection was added to the model, with blue-coloured responses to lock-ins demonstrating a positive loop for renewable integration (**R2 in** Fig. 6).

Another layer of this connection relates to the DSO’s flexibility operations. Congestion, particularly in high- and medium-voltage areas, has created opportunities to deploy demand-side flexibility where grid capacity is underutilized. DSOs are utilizing flexibility through incremental innovations in technology and market mechanisms, by rediscovering solutions based on the existing infrastructure and frameworks. However, challenges arise from the uncertainty and reliability of flexibility sources, the emerging nature of flexibility markets, and evolving regulatory frameworks (Fonteijn 2021). This underscores the need for improved TSO-DSO collaboration, as TSOs, responsible for balancing markets, influence and are influenced by congestion management efforts. For instance, flexibility procured for balancing might reduce its availability for congestion management, leading to operational conflicts if both markets demand flexibility simultaneously (Hadush and Meeus 2018). As one interviewee noted, “Right now, we don’t have a reliable way to predict grid availability, which makes it difficult to offer contracts that depend on spare capacity. It’s a challenge that will likely take years to address, as it requires close collaboration with the TSO. Historically, the TSO and DSO have operated separately, but now we’re beginning to work together—learning and understanding each other along the way.” (Int#10), which highlights an institutional reorientation between high- and low-voltage networks.

Incremental innovations in flexibility solutions, driven by entrenched congestion, have connected previously separate actors—high- and low-voltage grid operators, energy planners, households, and supply and distribution stakeholders—fostering a shared sense of urgency. The unintended consequences of these interactions and their implications are modelled in the following SD section.

#### **Rediscovering solutions: adaptive strategies for capacity management within grid constraints**

Faced with growing waiting lists and capacity constraints, the distribution grid operator has turned to incremental and adaptive innovations, *rediscovering solutions* within the existing grid infrastructure and path-dependent developments. These efforts aim to accelerate service for customers awaiting additional capacity or transitioning to electrified systems, such as medium-voltage clients requesting charging stations or grid reinforcements for electric vehicles. These innovations leverage locked-in mechanisms in two key ways: (i) technological responses—maximizing the efficiency of existing grid assets, and (ii) market responses—utilizing established market structures.

(i) ***Technology Responses***

Demand-side flexibility has become the primary innovation for optimizing the existing grid while awaiting infrastructure upgrades. Congestion pressures have pushed the grid operator to develop technology-driven solutions that reduce reliance on grid investments. As one participant noted, “If customers get on waiting lists and need to wait two years for grid capacity, businesses often find creative solutions to meet their electricity needs” (Int#4). Another added, “We now engage customers differently, asking if they can shift usage, like charging equipment at night. We’re developing smart solutions, such as time-based contracts (TCT), to enable grid access” (Int#9). These solutions are closely tied to the existing grid infrastructure, highlighting the importance of infrastructure interrelatedness.

By leveraging existing infrastructure more efficiently, smart solutions such as demand control and smart charging have emerged as path-dependent innovations, further reinforced by economies of scale as electric vehicle adoption rises. As one interviewee explained, “Smart charging is feasible, but the challenge is whether people will adopt it voluntarily or require enforcement. Stricter measures may be needed to distribute capacity more efficiently for electric vehicles” (Int#6). This approach was integrated into the model, forming a balancing loop for congestion **(B7).** Flexibility solutions demand greater customer cooperation, yet legal protections may lead to resistance. This *reorientation of synergies* has blurred traditional boundaries, as customers and grid operators now share the same problem space, fostering collaboration through bi-directional communication. However, this shift comes at a cost—under current legislation (Electricity Act 1998), DSOs bear congestion-related expenses, such as compensating curtailed wind farms, with these costs passed to society through grid taxes, exacerbating inequalities. Residential low-voltage consumers are disproportionately affected compared to larger commercial renewable energy producers. As one interviewee pointed out, “Windmill operators can choose where they want to connect to the grid, and they’re subsidized to do so. If their connection causes congestion and we have to curtail their windmills, we still have to compensate them, which is strange because we’re paying to get them on the grid and also paying when there is congestion…these costs are covered by society through tariffs, even though the issues stem from commercial parties” (Int#7).

**Fig. 7:**
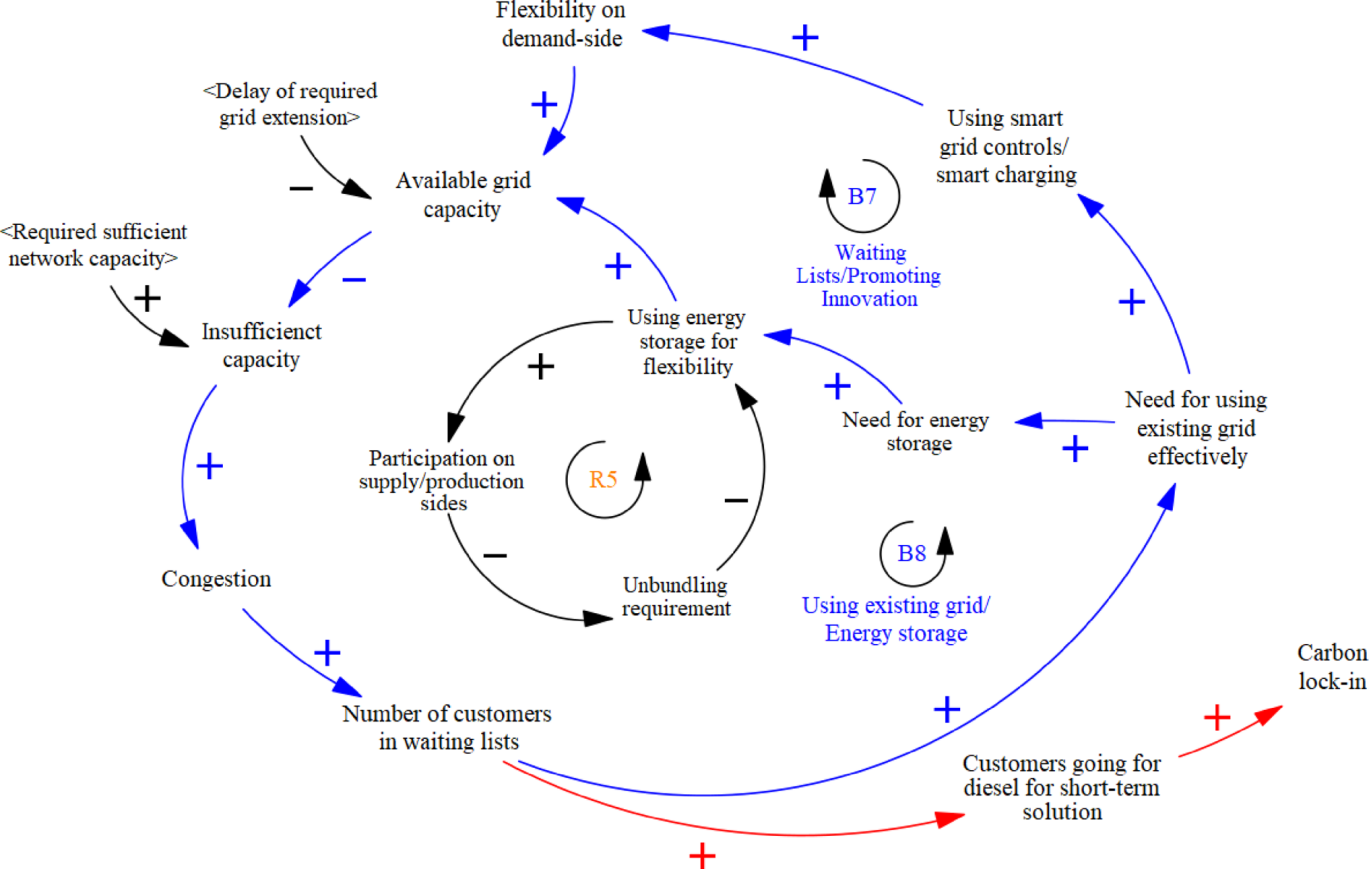
Technology-driven (Infrastructure) Exploration of Lock-ins

Experts also highlighted an unexpected consequence of waiting lists during the modelling: while the situation can encourage customers to change their demand patterns or invest in smart solutions, it may also lead to negative feedback, such as customers reverting to diesel aggregators or gas turbines to meet short-term energy needs of their business, reinforcing carbon lock-in. This dynamic is represented in the model with a red causal link (Fig. 7). As one interviewee explained, “There are also customers… if I can’t get my connections for renewables in that time period, let’s go back to fossil fuels, so again to carbon lock-in.” (Int#5).

As a regime-sustaining innovation, the experts mentioned the use of energy storage to increase the effective use of the existing grid, as represented in the model **(B8).** As participants noted, economies of scale in EVs and renewables present challenges: “Solar panels can charge electric cars, but there’s a mismatch: the sun shines mid-day, while charging demand peaks in the evening. Cars are typically at the office during the day, not home to store energy. Still, flexibility systems like optimized EV charging at warehouses and peak reduction for solar installations offer promising solutions” (Int#3).

This innovation also fosters *synergy* between energy system actors. However, Dutch law mandates full ownership unbundling of DSOs, barring them from energy generation, supply, or trade. This restriction limits their role in innovations like battery storage (**R5** in Fig. 7), reinforcing reliance on market parties. As one participant noted, DSOs must strictly separate energy supply and transport, while another added, “The downside is we’re often approaching highly demanding issues as if they’re free-market problems, with strict regulations on us but not on the customers and producers using the grid” (Int#7).

(ii) ***Market Responses***

**Fig. 8:**
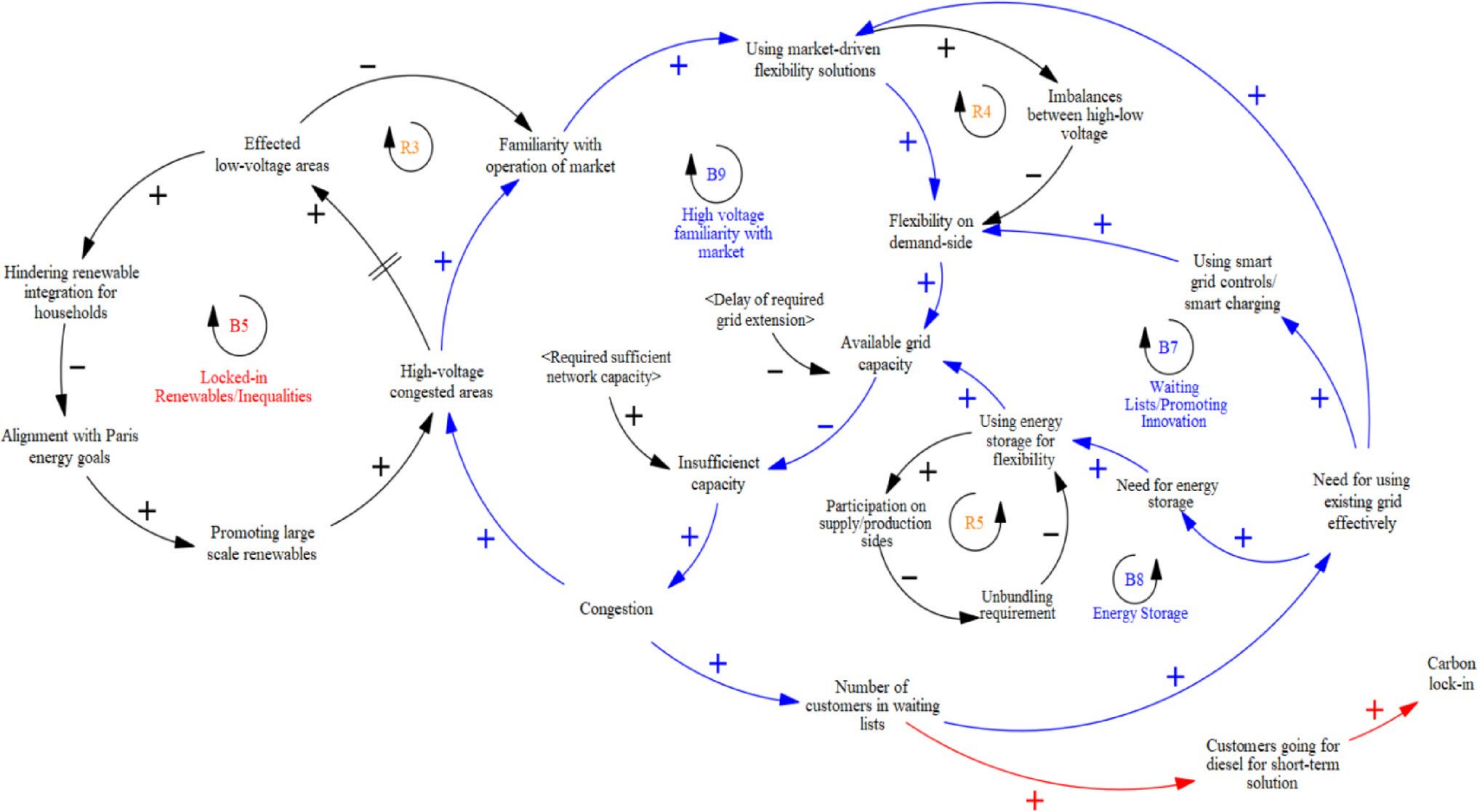
Market-driven exploration of lock-ins

Beyond technological innovations, leveraging existing regulatory and market mechanisms was highlighted as another important source of flexibility. Participants noted that high-voltage grids already incorporate flexibility, with larger customers accustomed to adjusting operations based on energy market stimuli, which represents the positive impact of market habituation. As one explained, “There are many customers… usually bigger ones. If they change their behaviour, the impact is big. Especially generators, who are already familiar with adjusting operations based on electricity market prices.” (Int#5). This has been added to the model during the second modelling session (**as B9 in** Fig. 8).

Despite the focus on incremental innovation through locked-in market mechanisms, a key concern is the disconnect between high- and low-voltage grids. Large energy consumers adapt flexibly, but households remain largely unaware. As one participant noted, “One challenge is that our customers currently don’t have much flexibility. Automation is limited, and it’s difficult for our customers, whether households or medium and large businesses. Many market participants aren’t very knowledgeable on this topic yet… on how to optimize their existing capacity.” (Int#7) Another added, “Bigger customers are used to adapting… whereas in the low-voltage grid, no one is really concerned with their electricity use, only with the bill.” (Int#5), as a reinforcing loop reflecting the limited market familiarity with low-voltage congested areas (**R3 in** Fig. 8). Raising awareness on congestion, demand-side management, and energy reduction was emphasized as a priority. “Many customers don’t realize how big the congestion issue is, or even understand what flexibility markets are, so they’re reluctant to participate. They also don’t see what’s in it for them.” (Int#9).

Moreover, not only from the customer point of view, but Interview findings highlight challenges in managing flexibility due to imbalances between transmission and distribution grids. These imbalances stem from risk predictions made earlier, which locked in standard load profiles (peaks in the morning and evening) for DSOs by the TSO, limiting dynamic grid allocation and flexibility now. As one participant explained, “Ten years ago, we didn’t coordinate with the transmission grid, but now we’re forced to. Still, we need each other. Transmission congestion means we’re told not to influence peak loads, even when more is possible. Customers shouldn’t wait ten years for capacity just because the transmission grid has a problem.” (Int#5). Constraints on nighttime capacity further complicate flexibility, as large clients sell capacity abroad. “With storage solutions like batteries, it seemed we could offer flexibility, but we’re limited because of transmission constraints… Extra capacity was allocated to energy traders, batteries, and large wind parks. This raises the question: Is it more valuable to supply 1,000, 2,000, or 10,000 small companies or just one or two large ones?” (Int#6), reinforcing the congestion problem due to those imbalances **(R4 in** Fig. 8). Ultimately, flexibility—whether through technology or market responses—requires a closer connection of organisational and institutional problem spaces in the energy transition.

**Fig. 9:**
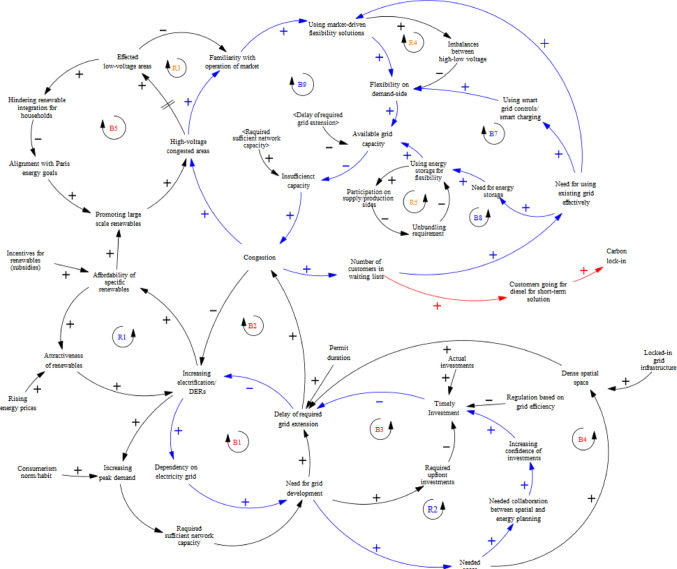
Final Model Incorporating Lock-in-Induced Responses

The final qualitative system dynamics model (Fig. 9) integrates expert feedback into the initial as-is system model (above Fig. 5), capturing the systemic consequences of existing governance frameworks on grid expansion delays, which form balancing loops that slow the energy transition. However, locked-in commitments to renewable energy and the urgency of sustainability transitions have also triggered counterbalancing mechanisms aimed at reducing congestion and accelerating grid adaptation. Expert feedback highlights that system dynamics modelling—through interviews, discussions, and visualized feedback—enhances understanding of decision consequences. Rather than focusing solely on technological solutions, this approach reveals the socio-technical dynamics underpinning grid capacity planning, identifying where lock-ins are already being leveraged and where further attention is needed to align sustainability strategies with operational realities.

## 5 Discussion

This paper investigates socio-technical lock-ins in electricity grid planning and network adaptation for renewable energy integration, focusing on a Dutch DSO facing ambitious decarbonization goals. It adopts a holistic perspective, exploring dynamic interrelations between technical and social elements rather than treating lock-ins as static events.

Using expert interviews and document analysis, the study identifies how economies of scale in distributed renewables, rising energy prices, and subsidy schemes have driven unexpected growth, straining the grid—especially in high- and medium-voltage segments. A century-old underground grid, not designed for high volumes of intermittent distributed energy, creates spatial constraints in an already dense network. Institutional sources of lock-ins, such as cost-efficiency-driven regulations, prioritize short-term efficiency over proactive grid investments, fostering a risk-averse organizational culture. The unbundled energy market structure has institutionalized the separation of production and distribution, creating significant information asymmetry that limits DSOs’ ability to anticipate developments on the production and supply side. Additionally, institutional learning around the “copper plate” principle—assuming unrestricted grid access—has reinforced a consumerist norm regarding grid capacity. This study has examined how interlocking feedback loops reinforce policy resistance, using systems thinking to navigate complexity.

However, it challenges the view that lock-ins are purely barriers to energy transitions. By focusing on a DSO as a regime actor, we show how internal lock-in mechanisms—embedded in infrastructure, regulatory routines, and organizational culture—interact with external system dynamics such as decentralized renewable production, rising electricity demand, and shifting societal expectations. The resulting friction generates pressures for adaptation, illustrating how regime actors mediate between entrenched routines and external demands. These dynamics echo Hughes’ concept of ‘reverse salients’—systemic bottlenecks arising from uneven development in large-scale systems (Hughes 1987). In our case, the grid’s inability to keep pace with renewable adoption has become a key bottleneck, prompting organizational adaptation and exposing tensions between legacy field logics (e.g., efficiency, reliability) and emerging sustainability logics (e.g., flexibility, coordination). As resource constraints like grid congestion intensify, lock-ins can reveal leverage points and open windows of opportunity for adaptation and innovation. In this way, the study advances socio-technical lock-in literature by showing how entrenched mechanisms can also create conditions for organizational change, aligning with emerging debates on lock-ins as potential leverage points rather than solely barriers (Goldstein et al. 2023). These insights have broader relevance for understanding lock-ins in sustainability transitions and for informing policy strategies in electricity grid planning.

### Lock-ins as catalyst for sustainability transitions

Sustainability transitions research has traditionally viewed lock-ins as obstacles that reinforce existing system trajectories. Examples include stranded fossil fuel assets hindering energy transitions (Brauers 2022), cognitive lock-ins in agri-food systems (Weituschat et al. 2022), and narrative lock-ins creating conflicts in circular economy transitions (Simoens and Leipold 2021). While this study acknowledges the constraining nature of lock-ins, it also examines how they can drive adaptive responses in grid operators’ practices. Distribution System Operators (DSOs), in particular, play a pivotal role in enabling energy transitions (Pereira et al., 2020; Netbeheer Nederland 2023), yet remain comparatively overlooked in transition studies. Responding to recent calls to explore how regime-embedded actors influence sectoral transitions (Fuenfschilling and Binz 2018; Grin 2020), this study focuses on a DSO to understand how it navigates between ambitious decarbonization targets and the operational realities of grid stability. In doing so, it also underscores the broader implications of DSOs’ adaptive practices for accelerating sustainability transitions.

In relation to RQ1, our findings show that socio-technical lock-ins fundamentally shape grid capacity planning by embedding DSOs in long-standing institutional, economic, and regulatory path dependencies. Analysing historical decision-making and systemic constraints, the research reveals how distribution grid planning has evolved under these path-dependencies as well as newer challenges from renewable energy integration. Historical cost-efficiency regulations, the “copper plate” principle of universal access, and entrenched investment routines have limited anticipatory action, resulting in delayed grid development and congestion. These dynamics illustrate not only how lock-ins create constraints for system expansion, but also the growing need for system integration, as DSOs must operate within an unbundled regime where supply and demand evolve rapidly and unevenly.

i. Turning to RQ2, the study shows that DSOs nonetheless navigate these lock-ins through adaptive practices. Rather than simply hindering transitions, these constraints can also open opportunities for adaptive behaviors that enable system-wide change. Framing infrastructure transitions as complex adaptive systems (Oughton et al. 2018), the study identifies three mechanisms through which lock-ins can catalyse innovation: Consequences of ***behavioural lock-ins***, particularly the long-standing reliability-focused culture within DSOs, have led to a ***organizational response*** of reframing congestion as an urgent and shared challenge rather than a temporary bottleneck. This awareness has driven internal restructuring, new KPIs, broader engagement with customers and policymakers on demand-side solutions, and raised new questions about the social responsibility of grid access.
ii. Consequences of ***institutional lock-ins***, rooted in the historically fragmented energy system, have facilitated ***relational response*** that reorient synergies across fragmented problem spaces, Grid constraints have strengthened collaboration between low- and high-voltage grid operators, municipalities, and spatial planners, aligning energy infrastructure with urban development, while also requiring new communication frameworks between customers and infrastructure providers.
iii. Consequences of ***technological lock-ins***, such as grid congestion and infrastructure delays, have driven ***technological response*** that rediscover solutions within existing infrastructures for capacity management. Instead of large-scale infrastructure expansion, the organisation has developed adaptive solutions, including demand-side flexibility, storage integration, and time-based contracts, based on the infrastructure interrelatedness and path-dependent incremental innovations.

Together, these findings show that what may appear as a “host of impediments” to capacity expansion can also stimulate adaptation when field logics collide. DSOs, positioned as regime actors, exercise agency within entrenched constraints by reconfiguring organizational practices, building relational bridges, and rediscovering technological solutions. In this sense, lock-ins are not fixed barriers but dynamic constraints that can evolve over time (Van Der Loos et al. 2020; Eitan and Hekkert 2023). While DSOs remain structurally restricted in their operational flexibility, the necessity of managing grid congestion has pushed them to move beyond demand-driven expansion toward more adaptive, supply-constrained planning. More broadly, this contributes to the transition literature by showing how regime actors, often treated as conservative, can also adapt tactically when confronted with interlocking constraints. The Dutch case is context-specific, but its complexity—rapid renewable growth, unbundled governance, and severe congestion—makes it *revelatory* of dynamics likely to surface elsewhere. The three adaptive mechanisms we identify can serve as a potential lens for analysing how regime actors adapt under pressure in other cases. DSOs across Europe face similar tensions between ambitious decarbonization targets, electrification trends, and grid capacity shortfalls. While particular responses will depend on national governance structures, market mechanisms, and policy regimes, the mechanisms observed here offer transferable insights into how entrenched conditions and transition pressures can trigger innovation and learning, and may invite other scholars to explore similar dynamics in different settings.

### Policy implications for electricity grid planning

The system dynamics approach is particularly useful in identifying not only the mechanisms that sustain grid capacity challenges but also the implications—both intended and unintended—of efforts to address them by visualising complexity behind, with interactions of multiple actors with competing priorities. While certain ‘unlocking’ mechanisms have emerged as presented in the previous section, they still require significant effort to become effective policy tools.

The analysis of institutional barriers and drivers in energy transition has primarily focused on high-level infrastructure expansion, such as transmission grid development (Tenggren et al. 2016), or the broader risks associated with scaling renewable energy (Nikas et al. 2020). However, this research shifts the focus toward the consequences of the energy transition for the distribution grid, which sits between transmission infrastructure and end consumers, making social and institutional aspects increasingly critical. In the Netherlands, the rapid increase in renewable energy integration has resulted in grid congestion and capacity limitations, delaying decarbonization efforts. Unlike previous studies that focus on preparing the grid for the transition, this study shifts the perspective to how the transition itself creates new constraints, particularly at the distribution level. While proposed flexibility solutions—such as flexibility markets (Villar et al. 2018), adaptive network tariffs (Bergaentzlé et al. 2019), and energy storage (Gür 2018)—aim to enhance resilience, they often overlook how DSOs navigate these constraints in practice. The key gap lies not in technical fixes but in understanding the institutional, technical, and behavioural barriers shaping DSO decision-making. This study addresses this by mapping sociotechnical interdependencies with system dynamics methodology.

Findings highlight the need for a more innovation-oriented culture within DSOs to accelerate incremental solutions like demand-side management and engage customers in flexibility markets. As capacity constraints reach low-voltage levels, public awareness and education on energy efficiency are crucial to mitigate demand pressures. Power asymmetries between high- and low-voltage networks raise policy questions about social prioritization—whether scarce capacity should go to a few large-scale users (e.g., data centres) or many smaller consumers. These decisions carry political implications, emphasizing the need for stronger TSO-DSO coordination and integration of energy and spatial planning for future uncertainties.

Regulatory adjustments are needed to allow system operators greater flexibility in risk assessment and alternative contracts that incentivize demand reduction. Currently, for many customers, the value of having (reserved) capacity is larger than ‘value of flexibility (incentives for reducing/shifting demand). Society must also reconsider whether the current level of reliability is necessary or if minor adjustments could optimize grid capacity. Companies, too, must take greater responsibility for flexible energy use—not just for their own benefit but for system-wide stability.

However, when flexibility is used to avoid capacity expansions, it can create future lock-ins by restricting electricity flow (Kuiken and Más 2019). As grids reach capacity, flexibility may become trapped locally, reducing system-wide adaptability. Therefore, regulatory frameworks should not only support flexibility-driven approaches but also accelerate grid expansion approvals, ensuring infrastructure development and demand-side solutions evolve together to address capacity challenges in the energy transition.

## 6 Conclusion and recommendations

Socio-technical transitions emerge from the interplay between infrastructures, institutions, and social systems, often reinforcing locked-in configurations that sustain unsustainable practices and hinder innovation. However, recent research increasingly focuses on unlocking these mechanisms to enable sustainability transitions. This study contributes to this agenda by examining the dynamic role of lock-ins in energy transition operations, demonstrating how they act as both constraints and catalysts for change.

Through a single case study of distribution grid capacity planning in the Dutch energy transition, the research addressed two questions: RQ1, by showing how socio-technical lock-ins embedded in regulatory routines, investment practices, and infrastructural path dependencies shape grid capacity planning and exacerbate congestion; and RQ2, by examining how DSOs navigate these constraints through adaptive practices. We identified three mechanisms: (i) reframing strategic, organizational questions with shared awareness on problems, (ii) reorienting institutional synergies by bridging previously siloed actors, and (iii) rediscovering solutions that enhance flexibility within existing infrastructure. These mechanisms illustrate how lock-ins can trigger organizational adaptation and innovation under conditions of scarcity. These findings highlight the critical but often overlooked role of DSOs as regime actors: positioned between ambitious decarbonization goals and operational realities, their responses offer insights into how entrenched systems adapt when field logics collide.

A key limitation of this study, inherent to system dynamics (SD) modeling, is its partial representation of system complexity, limiting full validation and transferability. Expanding data sources and quantifying causal loops could refine the model and provide deeper insights into feedback mechanisms. Nonetheless, SD effectively captures interdependencies and long-term implications, offering a valuable tool for decision-makers navigating sustainability transitions. In this sense, the Dutch case is particularly revelatory. Operating under a fully unbundled and liberalized electricity regime, the case DSO faces the dual challenge of rapid renewable integration and severe grid congestion within a densely populated and industrialized context. These conditions make visible how entrenched institutional, technological, and behavioural lock-ins collide with ambitious decarbonization pressures, creating both constraints and windows for adaptation. While responses will inevitably vary in countries with different institutional settings—strict ownership unbundling in the Netherlands versus DSOs still embedded in vertically integrated groups in Germany and France—our findings point to broader dynamics. The imbalance between infrastructure organizations’ internal constraints and the rapid growth of renewables and electrification shows how regime actors navigate tensions between structural inertia and transition pressures, often prompting adaptive responses under scarcity. We therefore see this study not only as context-bound but also as an invitation for comparative analyses of socio-technical lock-ins across electricity infrastructures and other utility systems. Exploring such dynamics across other infrastructure sectors would help clarify how lock-in conditions shape system interactions, where synergies between problem and solution spaces can be leveraged, and how incremental innovations can foster adaptability and resilience in sustainability transitions.

## Data Availability

The datasets generated during the analysis are publicly available through the link: (10.4121/91fe4b9d-49ee-4182-912d-0b0301f08eea.v1). The complete raw data (on interviews and confidential documents from the case organisation) is not available due to the nature of the data.
